# Health-related quality of life after ultrafocal salvage high-dose-rate brachytherapy for radiorecurrent prostate cancer: reporting the patient’s perspective

**DOI:** 10.1016/j.ctro.2020.10.002

**Published:** 2020-10-17

**Authors:** Marieke van Son, Evelyn Monninkhof, Max Peters, Jan Lagendijk, Jochem van der Voort van Zyp

**Affiliations:** a Department of Radiotherapy, University Medical Center Utrecht, Heidelberglaan 100, 3584 CX Utrecht, the Netherlands; b Department of Epidemiology, Julius Center for Health Sciences and Primary Care, University Medical Center Utrecht, Utrecht University, Heidelberglaan 100, 3584 CX Utrecht, the Netherlands

**Keywords:** Health-related quality of Life, Prostate cancer, Local recurrence, Focal therapy, Salvage treatment

## Abstract

**Purpose:**

For patients with a localized prostate cancer recurrence after radiotherapy, focal salvage treatment offers a less toxic alternative to whole-gland treatments, with the potential of preserving health-related quality of life (HR-QoL). With a focus on the patient’s perspective of treatment, this study aims to describe HR-QoL after ultrafocal salvage high-dose-rate brachytherapy (HDR-BT), and to explore predictive factors affecting HR-QoL.

**Material and methods:**

We included 100 patients treated with ultrafocal salvage HDR-BT. Prostate cancer-related HR-QoL was assessed by the EORTC QLQ-PR25 questionnaire. Domains were urinary symptoms, bowel symptoms and sexual activity/functioning. For each domain, a mixed effects model was made to estimate HR-QoL trends over time. For domains showing clinically relevant change (≥10 points difference), the mixed effects model was used to explore potential predictors (age, baseline HR-QoL score, T-stage, tumor location, CTV size, dose to organs at risk and history of ADT).

**Results:**

Median follow-up was 20 months (IQR 13–30). Mean questionnaire response rate was 86% (range 72–100%). Median baseline scores were 12 (urinary), 0 (bowel) and 67/50 (sexual activity/functioning). Urinary symptoms and sexual functioning showed clinically relevant deterioration over time (maximum difference of 11 and 12 points, respectively). Worse baseline score and higher administered dose to the urethra (≥16 Gy) were predictive of increased urinary symptoms (p < 0.01 and p = 0.03). Better baseline score was predictive of better sexual functioning (p < 0.01).

**Conclusion:**

Ultrafocal salvage HDR-BT has negligible impact on bowel symptoms but does affect urinary symptoms and sexual functioning. Lower impact is predicted for patients with favorable urinary and sexual function at baseline. Urethral dose constraints should be closely monitored.

## Highlights

• We analyzed patient-reported quality of life in 100 patients who underwent ultrafocal salvage HDR-brachytherapy.
• Patient-reported bowel symptoms were neglible.
• Urinary complaints increased and sexual functioning declined over time.
• Lower impact is predicted for patients with favorable function at baseline and lower dose to the urethra.

## 1 Introduction

As a result of treatment innovations and increased cancer survival, more attention is directed towards patient-reported outcomes such as health-related Quality of Life (HR-QoL). This trend is especially relevant for prostate cancer, with decreasing mortality rates in most countries despite increasing incidences [1].

Depending on tumor stage, prostate cancer recurrences occur in 10–50% of patients 10 years after external beam radiotherapy (EBRT) [2]. Most of these patients are treated with (delayed) androgen deprivation therapy (ADT) [3], which is a temporary suppressive treatment associated with significant side effects and deterioration of HR-QoL [4]. Although various whole gland salvage treatment modalities are available such as radical prostatectomy, low-dose-rate brachytherapy (LDR-BT), cryotherapy and HIFU, these are unpopular due to high failure and toxicity rates [5]. Although salvage prostatectomy and HIFU are associated with higher urinary incontinence rates (40–50%) than salvage cryotherapy or brachytherapy (7–12%), all modalities have high impotence (±75%) and urethral stricture rates (±20%), and 45–55% of patients experience a relapse after 4 years [6]. Whole-gland salvage irradiation causes toxicity by accumulation of dose to the surrounding organs at risk. Toxicity reduction is anticipated if the target is reduced from the whole gland to the tumor area alone. Since imaging advancements such as magnetic resonance imaging (MRI) and PSMA-PET/CT have improved detection of the exact tumor location, focal treatment is now clinically feasible [7], [8]. Reviews of the available literature on focal salvage treatments (including focal brachytherapy, HIFU and cryotherapy) have consistently shown that they are well tolerated with very limited severe genitourinary and gastro-intestinal toxicity (<5%) and with encouraging biochemical control rates (48–72% after 3 years) [9], [10].

The radiotherapy department at the University Medical Centre Utrecht has a 1.5T MRI high-dose-rate brachytherapy (HDR-BT) facility. Here, ultrafocal treatment of recurrent prostate cancer is performed by internal irradiation of the tumor under MRI-guidance. Due to the steep dose fall-off in brachytherapy, a high dose can be applied to the tumor while the surrounding healthy tissue receives low radiation exposure. It is therefore expected that patients experience less side effects and maintain their HR-QoL. Providing a detailed view on the patient’s perspective of this treatment, the current study aims to investigate prostate cancer-specific HR-QoL after ultrafocal salvage HDR-BT and to explore predictive factors that may impact HR-QoL.

## 2 Material and methods

### 2.1 Patients

Between July 2013 and March 2018, the first consecutive 100 patients with localized recurrent prostate cancer after primary radiotherapy were treated with ultrafocal salvage HDR-BT. Treatment was either performed within an institutional review board (IRB)-approved prospective study (Netherlands Trial Register [NTR] number 6123 or 7014) or outside the scope of a study protocol, including patients with higher-risk disease characteristics, such as seven patients with a solitary lymph node or bone metastasis who received upfront stereotactic radiotherapy. For study patients, trial inclusion criteria were PSA <10 ng/ml, PSA doubling time (PSADT) >12 months and MRI tumor stage ≤T2c (NTR 6123) or PSA ≤20 ng/ml, PSADT ≥9 months and MRI tumor stage ≤T3b (NTR 7014). All patients (on- or off-protocol) were prospectively followed in the same manner. Informed consent was obtained from all study patients. For patients treated off-protocol, the IRB waived the requirement for informed consent. For this report it was pragmatically chosen to analyze the first 100 treated patients, since they all had at least three months post-treatment follow-up before the start of the analysis.

### 2.2 Treatment

Using 3T multiparametric MRI (T2-weighted, diffusion-weighted and dynamic contrast enhanced sequences) and 68Ga-PSMA or 18F-Choline PET-CT, we delineated the gross tumor volume (GTV), clinical target volume (CTV, defined as five-millimeter margin around GTV) and organs at risk (OARs: bladder, rectum, and urethra). The rectum was delineated between the level of the sigmoid fold and the anal region, the bladder was delineated within the available field of view and the urethra was delineated one slice above and one slice under the prostate (sagittal plane). No PTV-margin was applied. Under the guidance of rigidly fused MRI/transrectal ultrasound images, MR-compatible catheters were transperineally inserted in and around the CTV. A subsequent 1.5T MRI scan was used for catheter reconstruction and adjustment of delineations. Radiation goal was to administera single dose of 19 Gy to 95% of the CTV. Dosimetry constraints were D1cc <12Gy for the bladder and rectum and D10% <17.7Gy for the urethra. With the radiation dose fully targeted at the CTV instead of a quarter or half of the gland, this treatment is generally described as ultrafocal.

### 2.3 Outcome assessment

HR-QoL was investigated using the EORTC QLQ-PR25 questionnaire, which was specifically designed for use among prostate cancer patients [11]. Questionnaires were sent out before treatment and after 1, 3, 6, 9, 12, 18 and 24 months, and yearly thereafter. The respective domains are urinary symptoms (9 items), bowel symptoms (4 items), sexual activity (2 items) and sexual functioning (4 items). As ADT was not part of this treatment, we did not analyze the domain hormonal treatment-related symptoms. For each item, patients were asked to indicate the extent to which they had experienced symptoms or problems during the past week (1: not at all, 2: a little, 3: quite a bit, 4: very much).

Domain scores were linearly transformed to a 0–100 scale if at least half of the items in the domain were answered. Higher scores either indicated more symptoms (urinary and bowel domains) or higher levels of functioning (sexual domains).

For the QLQ-PR25 questionnaire, no threshold value has been determined as the minimal clinically important difference in scores. In concordance with the QLQ-C30 questionnaire (HR-QoL of cancer patients in general, similar scoring range 0–100), we defined a change of ≥10 points as a clinically relevant difference [12].

### 2.4 Statistical analysis

To estimate average HR-QoL trends over time, a mixed effects model for repeated measures was made for each domain. Differences between baseline HR-QoL scores and follow-up time points were tested by adding time as a categorical variable to the mixed effects model. The significance level for HR-QoL change was set at p = 0.01, taking into account multiple testing. Trends were graphically displayed using the group least squares means and their standard errors.

Secondly, we explored potential predictors of change in HR-QoL within each model. This was only analyzed for the domains that showed clinically relevant change. We hypothesized that age, baseline HR-QoL score, T-stage of the tumor (as scored by the AJCC TNM eighth edition staging manual), size of the CTV and dose to the respective organs at risk (OAR) are potential factors predicting HR-QoL change. For the sexual domain, we also included previous use of ADT and dorsolateral location of the tumor. Assessment of the relation between these predictors and change in HR-QoL was performed in a multivariable model, calculating odds ratios and their 95% confidence intervals (CI). Predictors with p-values <0.05 were considered statistically significant.

Statistical analyses were performed with R statistical software (version 3.5.1; the R foundation for Statistical Computing, Vienna, Austria) and IBM SPSS statistics (version 23.0).

## 3 Results

The median follow-up time was 20 months (interquartile range [IQR] 13–30 months). Questionnaire response rates were between 72% (3 months) and 100% (36 months), with a mean response rate of 86%. At baseline, patients reported mild urinary symptoms (median score 12, IQR 8–21) and negligible bowel symptoms (median score 0, IQR 0–8). Sexual activity was at a baseline median score of 67 (IQR 50–83), and sexual functioning at median 50 (IQR 42–67). The median CTV D90% was 21.43 Gy (IQR 19–21.5), with a median of 9 brachytherapy catheters used for the implant (IQR 8–11). A summary of baseline patient and tumor characteristics is displayed in Table 1.

**Table 1:** Baseline characteristics (n = 100).

			Median (IQR) or percentages
Age (years)			71 (67 – 74)
Primary treatment	EBRT		53%
	LDR-BT		44%
	Whole-gland HDR-BT		1%
	Ultrafocal HDR-BT		2%
History of ADT*	No		80%
	Yes, neo-adjuvant		6%
	Yes, adjuvant		14%
TNM-stage on imaging	T	T2	62%
		T3	36%
		T4	2%
	N	N0	96%
		N1	4%
	M	M0	97%
		M1	3%
Size of the CTV (cc)			10 (7 – 16)
Baseline quality of life scores^	Urinary symptoms		12 (8 – 21)
	Bowel symptoms		0 (0 – 8)
	Sexual activity		67 (50 – 83)
	Sexual functioning		50 (42 – 67)

Legend: IQR: interquartile range, EBRT: external beam radiotherapy, LDR-BT: low-dose-rate brachytherapy, HDR-BT: high-dose-rate brachytherapy, ADT: androgen deprivation therapy, TNM-stage: tumor/node/metastasis stage, CTV: clinical target volume.

* As part of primary treatment.

^ EORTC QLQ-PR25, scale 0 – 100.

**Fig. 1:**
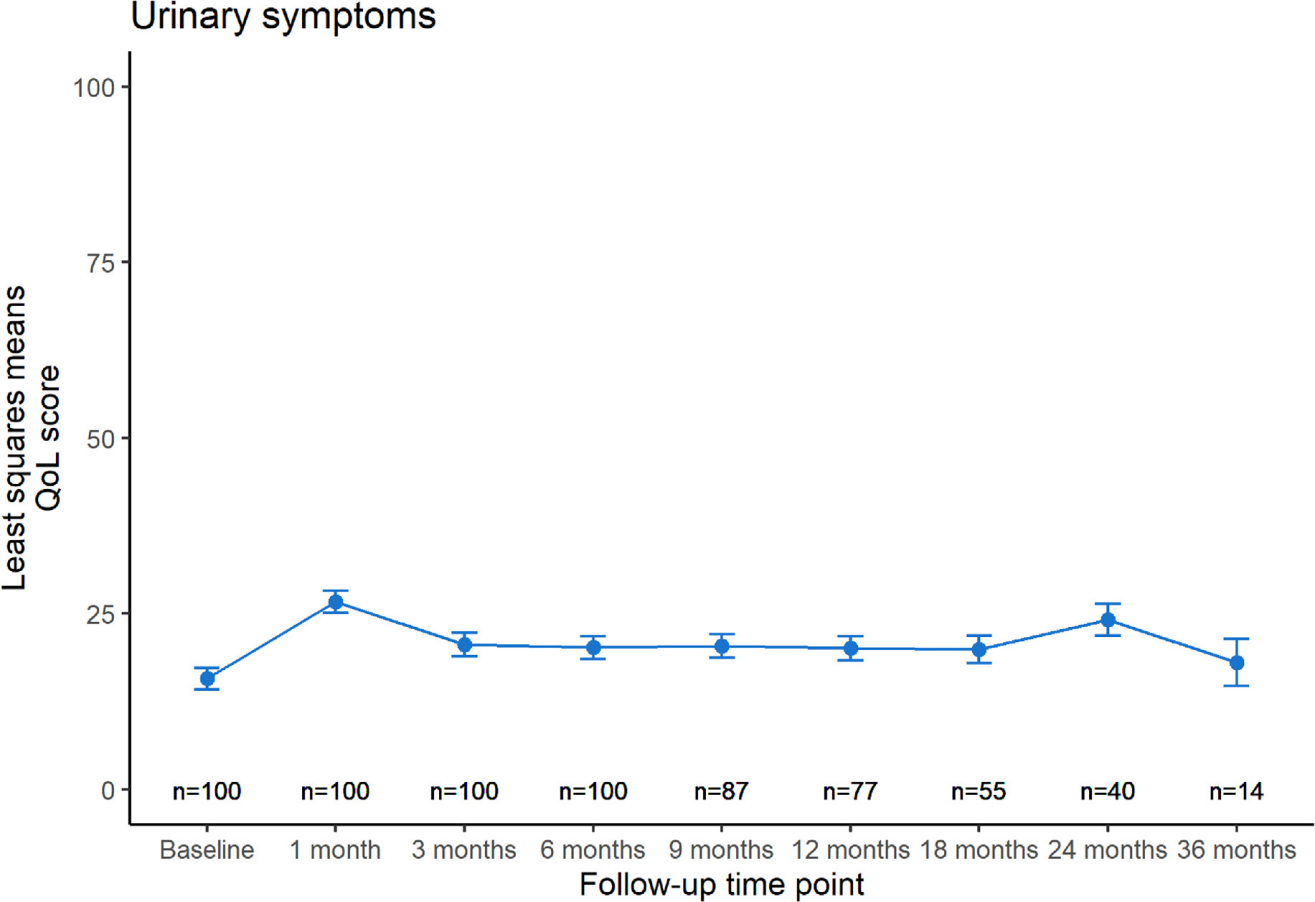

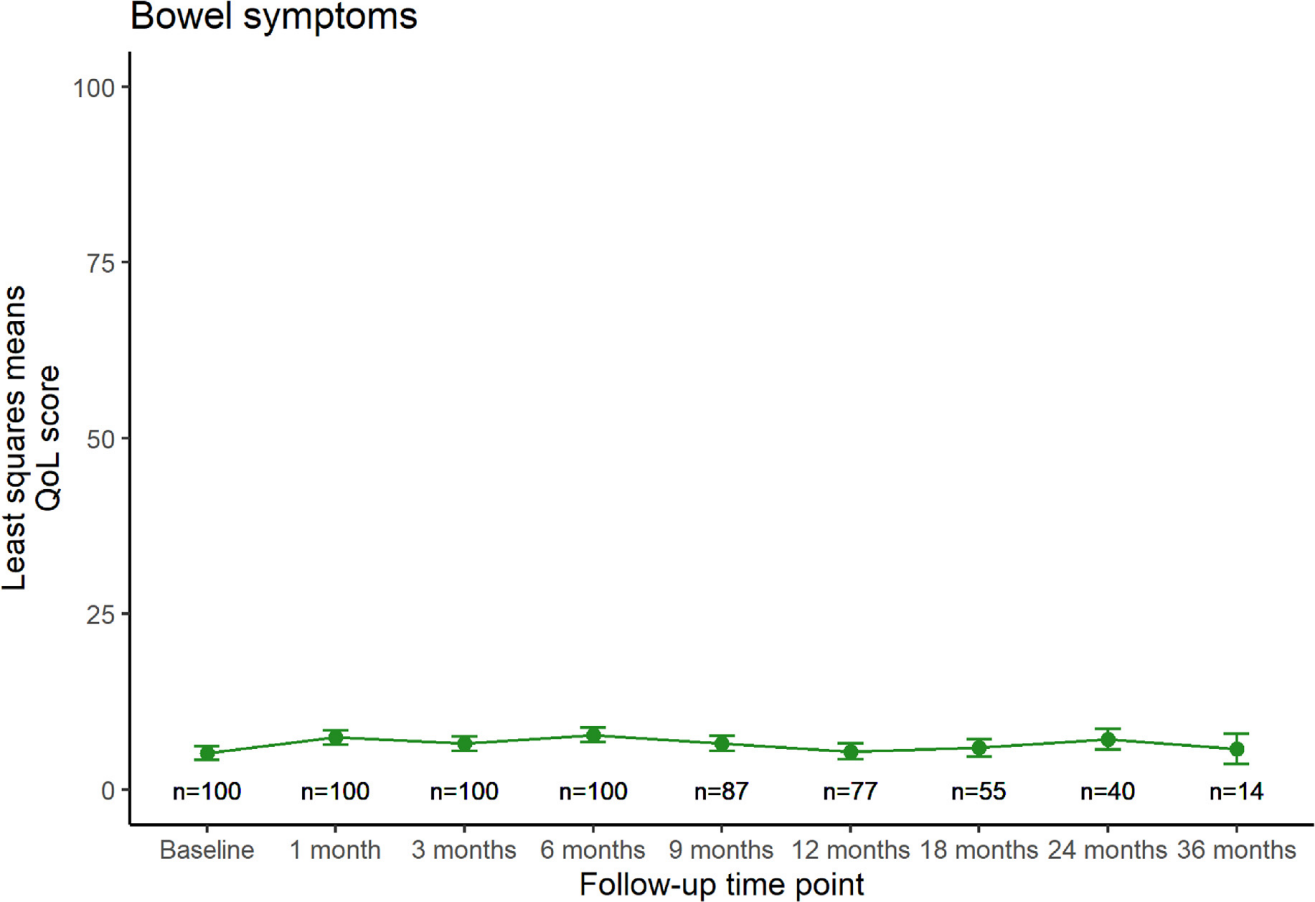

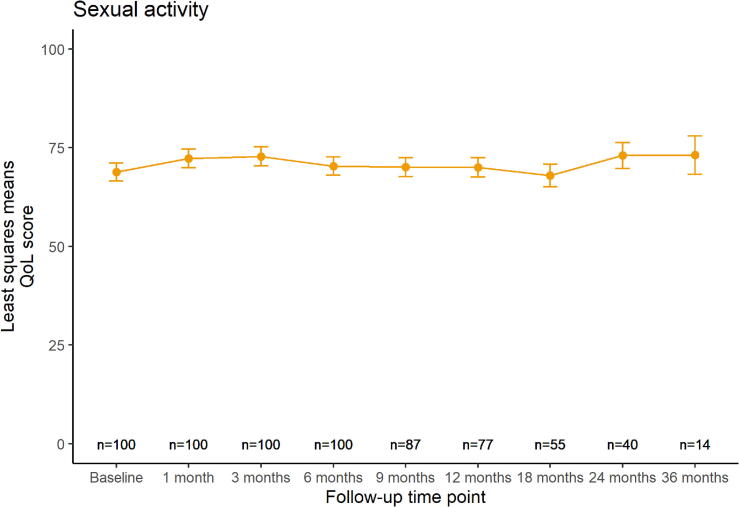

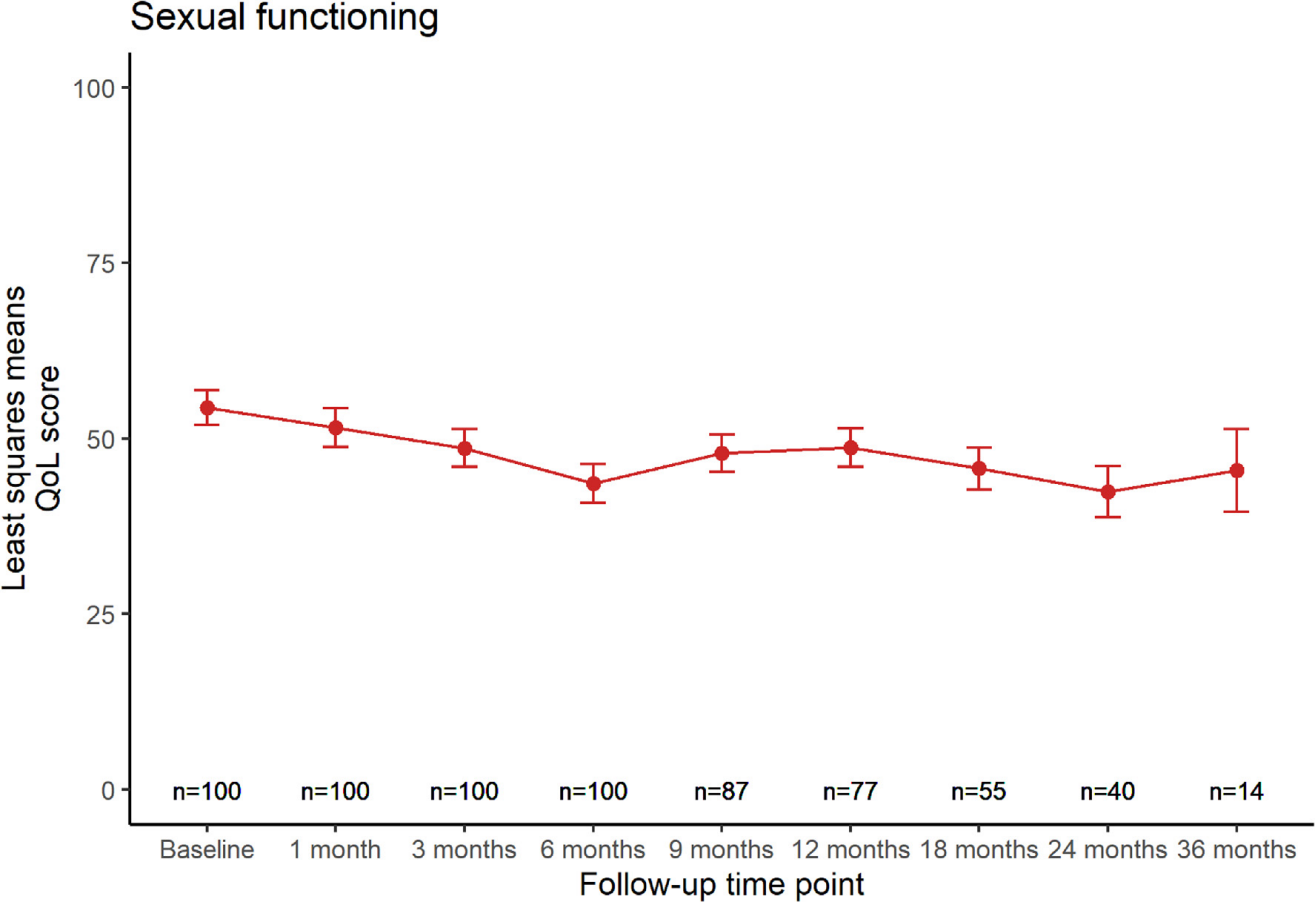
a,b,c,d: Modeled quality of life trends over time. Least squares means are displayed with their standard errors at each follow-up time point. Number of patients at risk who received a questionnaire at each time point is displayed on the bottom.

Fig. 1 shows the modeled quality of life trends over time for each HR-QoL domain, displaying least squares means with their standard error (SE) at each follow-up time point. Urinary symptoms (Fig. 1-a) increased with +11 points in the first month after treatment (p < 0.01). Afterwards, the score recovered almost completely back to baseline level (least squares mean difference of 2 points between baseline and 36 months follow-up, p = 0.5). Bowel symptoms (Fig. 1-b) remained stable at a lower level over time, with a maximum least squares mean difference of +3 points at 6 months (p = 0.04). Sexual activity (Fig. 1-c) showed a similar stable trend, with a maximum least squares mean difference of +4 points at 3 months (p = 0.1). Sexual functioning (Fig. 1-d) showed a downward trend over time, with a temporary recovery between six and twelve months, but with a maximum least squares mean difference of −12 points at 24 months (p < 0.01).

An explorative assessment of potential predictors for HR-QoL change was performed for the domains urinary symptoms and sexual functioning, as these domains showed clinically relevant change over time (Table 2). Higher (i.e. worse) baseline HR-QoL score and higher administered dose to the urethra were significant predictors for urinary symptoms. A post-hoc cut-off analysis revealed that a constraint of 16 Gy was the lowest value at which urethra D10% was a significant predictor in the model. Higher (i.e. better) baseline HR-QoL score (p < 0.01) was predictive of better sexual functioning over time.

**Table 2:** Association of predictors with HR-QoL change per affected domain.

Domain			95% CI		
		β	lower	upper	p-value
Urinary symptoms	Administered dose to the urethra	0.62	0.19	1.06	*<0.01*
	Administered dose to the bladder	−0.47	−1.03	0.09	0.1
	Baseline HR-QoL score	0.73	0.59	0.87	*<0.01*
	Age	−0.03	−0.35	0.28	0.83
	Size of the CTV (cc)	−0.09	−0.36	0.18	0.52
	Tumor stage on MRI >T2	3.76	−0.74	8.78	0.2
Sexual functioning	Previous use of ADT*	7.15	−1.2	15.5	0.07
	Baseline HR-QoL score	0.68	0.48	0.88	*<0.01*
	Age	−0.26	−0.9	0.37	0.42
	Size of the CTV (cc)	0.05	−0.34	0.44	0.81
	Tumor stage on MRI >T2	−5.17	−12.22	1.88	0.15
	Dorsolateral location of the tumor	5.09	−2.86	13.04	0.21

Legend: HR-QoL: health-related quality of life, CTV: clinical tumor volume, ADT: androgen deprivation therapy, β: estimate of the effect, 95% CI: 95% confidence interval.

* As part of primary treatment (neo-adjuvant or adjuvant).

A more detailed view on individual HR-QoL items is provided in Supplementary Table 1, with separate item scoring patterns. The table displays the percentage of patients reporting any symptom (score > 1) at each follow-up time point. Most reported urinary symptoms in the first month after treatment were dysuria, urgency, difficulty leaving the house and being limited in daily activities. Incontinence was also frequently reported, with a maximum of 14 patients declaring the need to wear an incontinence aid. Sexual functioning was mainly impaired by erectile dysfunction, ejaculation problems and sexual intimacy issues.

## 4 Discussion

This study shows that ultrafocal salvage HDR-BT has limited effect on patient-reported bowel function and sexual activity but causes a (temporary) increase in urinary symptoms and a decrease of sexual functioning over time. Predictive factors for deterioration of urinary HR-QoL are increased urinary symptoms at baseline and higher administered dose to the urethra (≥16 Gy). Higher level of baseline sexual functioning was predictive of better sexual HR-QoL.

A comparison with our previous work on ultrafocal salvage HDR-BT shows similar trends in terms of treatment-related toxicity. Our first report (n = 17, median follow-up 10 months) described physician-graded toxicity following the Common Terminology Criteria for Adverse Events version 4.0 (CTCAE). There was minor grade 1 rectal toxicity (mild or asymptomatic) and urinary toxicity was limited to approximately 25% grade 2 (moderate) and 5% grade 3 (severe) toxicity. Grade 3 new-onset erectile dysfunction occurred in 1/6 patients with full erectile function at baseline and 1/7 patients with moderate erectile dysfunction at baseline [13].

In a more recent update (n = 50, median follow-up 31 months), 4% had new-onset grade 2 rectal toxicity. While severe urinary toxicity was still limited (2%), more patients had developed grade 2 toxicity (52%). Grade 3 new-onset erectile dysfunction was seen in 22%. Regarding patient-reported toxicity, the IPSS revealed a temporary increase of urinary symptoms in the first month after treatment (maximum median score 11.5). The International Index of Erectile Function (IIEF) showed a downward trend of erectile function over time, from median score 11 at baseline to median 3 after 3 years follow-up [14].

Within the current literature on salvage treatments for radiorecurrent prostate cancer, there is limited data of patient-reported HR-QoL. Reports of HR-QoL that have been published are heterogeneous, using a variety of different questionnaires. Two studies reported IPSS and IIEF scores after whole-gland salvage treatments, namely whole-gland salvage LDR-BT (n = 19) and whole-gland salvage HIFU (n = 81). The questionnaires revealed a peak to moderate urinary symptoms (mean IPSS ±15) and a deterioration to severe ED (mean IIEF ±6) in the first year [15], [16]. Another study on whole-gland salvage HIFU in 61 patients used the University of California Los Angeles Prostate Cancer Index (UCLA-PCI) as patient-reported outcome measurement. They reported clinically significant urinary and sexual function deterioration after 1.5 years follow-up. At a scoring range of 0–100, mean urinary function decreased with 12 points (p < 0.01) and mean sexual function decreased with 15 points (p < 0.01). Bowel function was not affected [17].

Regarding targeted salvage treatments, only two studies described HR-QoL. A study on ultrafocal salvage LDR-BT (20 patients) used the EORTC QLQ-PR25, reporting a clinically significant increase of median 12 points in urinary symptoms after 3 years (p < 0.01) [18]. A study on focal salvage HDR-BT to a quadrant of the prostate (15 patients) used the Expanded Prostate Cancer Index Composite (EPIC). They reported a significant deterioration of sexual function after 3.5 years (approximately −20 points on a 0–100 scale, p < 0.01), whereas the urinary and bowel domains were not significantly affected. The median IPSS never exceeded a score of 10 [19].

To have a better understanding of what factors predict HR-QoL, we explored the association between several predictors and HR-QoL change. The apparent predictors for the urinary domain confirmed our expectations. We already screen for baseline urinary symptoms using the IPSS questionnaire, with scores >15 being a contra-indication for treatment. Although severe urinary toxicity has been low, we are strict in adhering to our urethral dose constraint (17.7 Gy) to account for the more frequently occurring moderate urinary symptoms. Following our cut-off analysis, a lower constraint might be an improvement.

For the sexual functioning domain, surprisingly age was not a significant predictor for HR-QoL deterioration, showing that sexual functioning varies among men of similar ages. Interestingly, the level of sexual activity did not seem to be affected over time.

It has been suggested that substantial radiation dose to the dorsolaterally situated neurovascular bundles (NVBs) may cause erectile dysfunction [20]. Although we expect the dose to the NVBs to be relatively low with ultrafocal HDR-BT, 87/100 patients had a dorsolaterally located tumor, of which 25% was bilateral. Due to a lack of clear guidelines on identification and delineation of the NVBs, we were not able to directly assess the relation with NVB received dose.

Although outside the scope of this patient-reported outcome study, a recent comparative trial has raised concerns about the oncological effectiveness of a single-dose HDR-BT regimen in the primary setting. This trial randomized 170 patients between whole-gland 1x19Gy and 2x13.5Gy and reported 5-year biochemical control rates of 73.5% (single-dose) versus 95% (two-fraction) [21], with similar low morbidity [22]. Unfortunately, there is no comparative data available on single-dose versus two-fraction focal salvage HDR-BT. It is therefore too early to suggest that this translates to the (focal) salvage setting.

A limitation of this study is the relatively short follow-up time. Although it is not expected, late treatment effects from delayed radiation damage may cause more HR-QoL deterioration in the future. Strengths of this study include the prospective nature, the high questionnaire response rates and the large patient group included in the analysis.

## 5 Conclusions

In conclusion, ultrafocal salvage HDR-BT seems to have a transient effect on patient-reported urinary function and no clinical effect on patient-reported bowel function. While sexual activity does not seem to decrease, patients report a deterioration of sexual functioning over time. Patients with impaired function at baseline (increased urinary symptoms or decreased sexual functioning) may have a higher risk of domain-specific HR-QoL deterioration over time, showing the importance of symptom assessment before treatment. Radiation dose to the urethra should be kept at a minimum to avoid urinary symptoms after treatment. This information may be used to improve treatment planning and patient counseling before treatment.

## 6 Disclosure statement

M.J. van Son: nothing to disclose.

E.M. Monninkhof: nothing to disclose.

M. Peters: received a grant by the Dutch Cancer Sociey (KWF Kankerbestrijding) [grant number 10932].

J.J.W. Lagendijk: nothing to disclose.

J.R.N. van der Voort van Zyp: received a grant by the Dutch Cancer Sociey (KWF Kankerbestrijding) [grant number 10932].

## Funding

This work was supported by the Dutch Cancer Sociey (KWF Kankerbestrijding) [grant number 10932]. They had no role in the collection, analysis and interpretation of data, in the writing of the report or in the decision to submit the article for publication.

## Declaration of Competing Interest

The authors declare that they have no known competing financial interests or personal relationships that could have appeared to influence the work reported in this paper.
