# Supplementary material for: Health-related quality of life after ultrafocal salvage high-dose-rate brachytherapy for radiorecurrent prostate cancer: reporting the patient’s perspective
Source: Clin Transl Radiat Oncol. 2020 Oct 17;25:81–7. doi: 10.1016/j.ctro.2020.10.002 (PMC7586050; doi:10.1016/j.ctro.2020.10.002)
Supplement: Supplementary data 1 [file mmc1.docx]

**Appendix**

| **Supplementary table 1 – Presence of symptoms per HR-QoL scoring item**  **(EORTC QLQ-PR25)** | | | | | | | | | |
| --- | --- | --- | --- | --- | --- | --- | --- | --- | --- |
|  | Baseline | 1 month | 3 months | 6 months | 9 months | 12 months | 18 months | 24 months | 36 months |
| No. patients | 100 | 100 | 100 | 100 | 87 | 77 | 55 | 40 | 14 |
| **Urinary symptoms** |  |  |  |  |  |  |  |  |  |
| Urinary frequency | 79 | 85 | 76 | 75 | 81 | 81 | 77 | 71 | 64 |
| Nocturia | 71 | 76 | 75 | 70 | 71 | 77 | 75 | 72 | 79 |
| Urinary urgency | 60 | 76 | 69 | 67 | 70 | 73 | 77 | 69 | 79 |
| Sleeping disturbances | 32 | 42 | 36 | 33 | 26 | 36 | 29 | 56 | 21 |
| Difficulty leaving the house | 20 | 36 | 28 | 30 | 36 | 30 | 26 | 39 | 14 |
| Urinary incontinence | 21 | 43 | 38 | 42 | 40 | 41 | 42 | 40 | 36 |
| Dysuria | 3 | 41 | 15 | 17 | 16 | 10 | 10 | 8 | 0 |
| ***If wearing an incontinence aid:*** |  |  |  |  |  |  |  |  |  |
| Problems with incontinence aid | 17 | 23 | 22 | 43 | 46 | 38 | 33 | 50 | 50 |
| ***No. responses*** | ***6*** | ***13*** | ***9*** | ***14*** | ***11*** | ***8*** | ***9*** | ***6*** | ***4*** |
| Limited in daily activities | 13 | 25 | 18 | 27 | 26 | 25 | 23 | 38 | 15 |
| **Bowel symptoms** | | | | | | | | | |
| Limited in daily activities | 13 | 24 | 20 | 24 | 19 | 17 | 21 | 21 | 15 |
| Fecal incontinence | 13 | 15 | 12 | 17 | 16 | 11 | 10 | 14 | 21 |
| Rectal bleeding | 11 | 18 | 15 | 15 | 12 | 8 | 2 | 6 | 7 |
| Abdominal bloating | 18 | 27 | 26 | 23 | 24 | 21 | 23 | 31 | 7 |
| **Sexual activity and functioning** | | | | | | | | | |
| Sexual desire | 80 | 72 | 76 | 76 | 78 | 74 | 77 | 74 | 71 |
| Sexually active | 64 | 53 | 60 | 57 | 63 | 60 | 60 | 62 | 36 |
| ***If sexually active over last 4 weeks:*** |  |  |  |  |  |  |  |  |  |
| Satisfaction sexual experience | 98 | 94 | 92 | 100 | 95 | 95 | 93 | 95 | 83 |
| Erectile dysfunction | 80 | 88 | 92 | 98 | 90 | 90 | 90 | 90 | 100 |
| Ejaculation problems | 71 | 71 | 78 | 85 | 81 | 82 | 83 | 79 | 100 |
| Sexual intimacy problems | 41 | 55 | 58 | 51 | 60 | 54 | 55 | 61 | 67 |
| ***No. responses*** | ***50*** | ***31*** | ***39*** | ***37*** | ***40*** | ***39*** | ***29*** | ***18*** | ***6*** |
| Legend: percentage (%) of patients with score >1.  For each individual scoring item, patients reported symptoms or problems during the past week, ranging between 1: not at all, 2: a little, 3: quite a bit, and 4: very much. | | | | | | | | | |
